# Crossing Kingdoms: How Can Art Open Up New Ways of Thinking About Science?

**DOI:** 10.3389/fbioe.2020.00715

**Published:** 2020-07-03

**Authors:** Erika Szymanski, Tarsh Bates, Elise Cachat, Jane Calvert, Oron Catts, Lenny J. Nelson, Susan J. Rosser, Robert D. J. Smith, Ionat Zurr

**Affiliations:** ^1^Department of English, Colorado State University, Fort Collins, CO, United States; ^2^SymbioticA, University of Western Australia, Perth, WA, Australia; ^3^UK Centre for Mammalian Synthetic Biology, The University of Edinburgh, Edinburgh, United Kingdom; ^4^Science, Technology, and Innovation Studies, The University of Edinburgh, Edinburgh, United Kingdom

**Keywords:** responsible research and innovation, art-science collaboration, interdisciplinarity, synthetic biology, hybrid taxa

## Abstract

“Crossing Kingdoms” is an artist-led experiment in the biological fusion of mammalian and yeast cells and the cultural discussions of these phenomena. We present this collaboration as an experiment in responsible research and innovation (RRI), an institutionalized format for ensuring that researchers reflect on the wider social dimensions of their work. Our methods challenged us as researchers to reflect on interdisciplinary collaboration and the possibility of innovating in biology for artistic purposes, challenged audiences to reflect on biological boundaries, and challenged both groups to reflect on what it means to be responsible in science. We conclude that our experiment in RRI was successful because we have asked unexpected questions—a contrast to RRI implemented as a standard protocol. Our experiment has implications for biologists and artists pursuing interdisciplinary collaborations with each other and for researchers thinking about implementing RRI as more than a box-ticking exercise.

## Introduction

Lewis Thomas, in 1974, called cell fusion “the most unbiologic of all phenomena, violating the most fundamental myths of the last century, for it denies the importance of specificity, integrity, and separateness in living things” (Thomas, 1974). We might likewise call interdisciplinarity the most unacademic of all phenomena, violating fundamental myths about generating quality research by denying the essentiality of narrow disciplinary expertise (Apostel, 1972). Yet much has changed in the half-century since 1974. Both cell fusion and interdisciplinarity have become increasingly popular and mundane.

Twenty-first century researchers challenge boundaries—among species, and between living things and machines, for example—that inhabitants of the twentieth century tended to take for granted. Simultaneously, twenty-first century researchers, largely in natural sciences and engineering, are being asked to reflect on how their work ramifies through society. That expectation of reflection has been institutionalized as responsible research and innovation (RRI) in Europe and, increasingly, elsewhere. Definitions of RRI by social scientists tend to emphasize collective care—for example, Stilgoe, Owen, and Macnaghten’s definition of responsible innovation as “taking care of the future through collective stewardship of science and innovation in the present” (Stilgoe et al., 2013). However, RRI is often implemented as a checklist through which researchers demonstrate that they have considered social implications of their research—a demonstration that quickly becomes formulaic.

We report here on an experiment in RRI, “Crossing Kingdoms,” (The Tissue Culture and Art Project, 2018) that challenges cell and disciplinary boundaries while exploring the boundaries of what it means to be responsible with biology. “Crossing Kingdoms” is an artist-led experiment in the biological fusion of mammalian and yeast cells and cultural discussions of these phenomena, and a social science experiment in RRI. It is also an experiment in biology for artistic purposes. Here, we describe the project, the methods of its development, and why we see it as a successful experiment in RRI.

“Crossing Kingdoms’” aims to make a biological entity—a yeast-mammalian hybrid—that would not arise without human intervention, and to exhibit the making of that entity to provoke the question: what is this living thing and what consequences follow its existence? The project brings together artists, synthetic biologists, and social scientists, who all approach this primary aim with different questions. The central artistic questions the project investigates are: How do multi-kingdom cell fusions challenge categories and understandings of life? Where do they belong in biological and wider cultural classifications? The biological question began as: can we use a heterologous protein from a snake virus to fuse cells that would not ordinarily fuse? The social scientists began by asking: what happens in an art-science project led from the outset by the artists? And what makes an exercise in RRI responsible? As with many collaborations, however, we began with shared curiosity and subsequently identified complementary research questions.

Barry, Born, and Weszkalnys (themselves social scientists) characterize art-science collaborations as following logics of accountability, innovation, or ontology (Barry et al., 2008). Logics of accountability use art to make science more palatable to popular audiences, or to ask ethical or other critical questions through art such that the science itself can carry on unaffected. Logics of innovation use artists to infuse creativity into technical solution-building. Logics of ontology, in contrast, try to place artists and scientists on level footing so that ways of thinking from art can come into science and vice-versa. “Crossing Kingdoms” was built on a logic of ontology. We present this schema to make explicit that our goals have never been to educate popular audiences through art, nor to use art to advance science.

## Disciplinary Fusion

### Constructing the Collaboration

**FIGURE 1:**
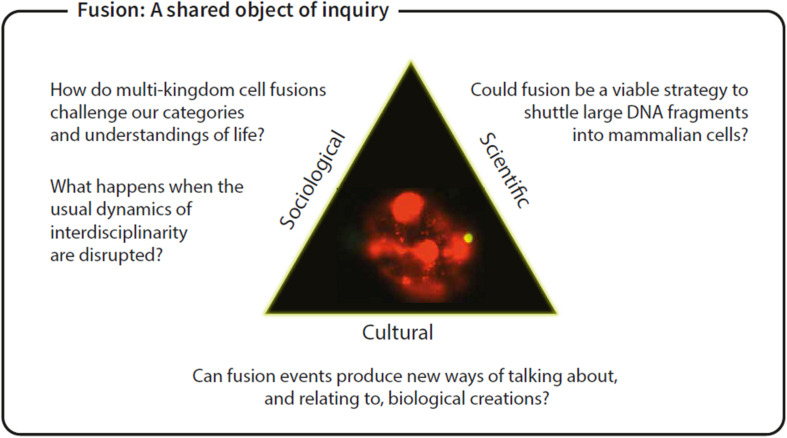
Fusion: A shared object of inquiry.

Crossing Kingdoms began with a dragon. Several of us attended the SB7.0 conference in Singapore in 2017, at which Alina Chan, a post-doctoral researcher at Harvard, introduced human artificial chromosomes (HACs) for large DNA delivery by suggesting that she was ultimately interested in making dragons (SB7.0 – Alina Chan, 2017). Discussing her presentation in the tea break that followed, we discovered shared interests in cross-kingdom cell fusions that yield new kinds of perhaps-unclassifiable living things (Figure 1).

After returning to our respective institutions, the artists suggested to the social scientists that we collaborate to explore building such fusions. A grant was written and funded, led by the artists, who traveled from Australia to Edinburgh to meet synthetic biologists with enabling technical skills. The grant aimed to promote interdisciplinary and international networking; it could be funded with a strong conceptual idea but no specific technical plan. A technical plan crystallized when a scientist unfurled a poster on the floor of an empty classroom and everyone—artists, social scientists, and synthetic biologists—excitedly joined in conversation around an image of fusing cells. Learning more, the artists became intrigued by the poetry of mammalian cells compelled to fuse with each other because they had been genetically modified with a snake virus protein. Their questions led to developing an experimental strategy that connected with their artistic goals.

Fitzgerald and Callard (2015) argue that such interdisciplinary entanglements enable “awkward intra-disciplinarity” in which project members from different disciplines work with no pre-established structure so that everyone becomes mixed up in the work and what comes out of it. Such awkward intra-disciplinarity contrasts with structured interdisciplinarity in which each member’s contribution is predetermined. In our case, waiting to construct specific experimental plans until after the collaboration was underway enabled the project team to explore with each other on equal footing without artificially curtailing each member’s role.

Our method of disciplinary fusion differs from many art-science collaborations in being guided by artistic research interests, rather than by goals to promote scientific research to broader audiences (Barry, Born, and Weszkalny’s logic of accountability) (Barry et al., 2008). The artists contributed initial ideas and goals, wet lab work, image capture, and final gallery presentations. The synthetic biologists contributed cell fusion technology and expertise, lab space and materials, and wet lab work. The social scientists became the glue holding the collaboration together, facilitating contacts, helping the project find institutional space, and driving an additional layer of analysis to make sense of the collaboration. Everyone has benefited from exchanging ideas that have subsequently fed additional research, collaboratively and in our “home” disciplines. These roles were identified through working together, not *a priori*.

Importantly, we built on pre-existing relationships. We cannot overemphasize how important mutual trust and understanding are to the success of our collaboration, or how important long-term relationships are to cultivating and maintaining that trust and understanding.

### Artistic Explorations

Our process-led artistic research methods have joined artistic exploration and laboratory work to recontextualize scientific experiments. Although biological research involves affective relations—empathy and other emotions—among scientists and research objects, typical scientific communication systematically erases those relations; scientific knowledge becomes explicitly impersonal so that it could, in theory, be produced by anyone anywhere. Art illuminates that usually hidden personal dimension so that wider audiences can engage with how research intersects with what it means to be human. Moving biological techniques and research outputs from research laboratories to public art galleries can make the familiar strange again, and enable thinking—or feeling, or viscerally reacting—in fresh ways. Art thus opens up otherwise unconventional experiences of biotechnologies and provides opportunities for broader discussions about assumptions, implications, values, and risks in these technologies and the boundaries they challenge.

**FIGURE 2:**
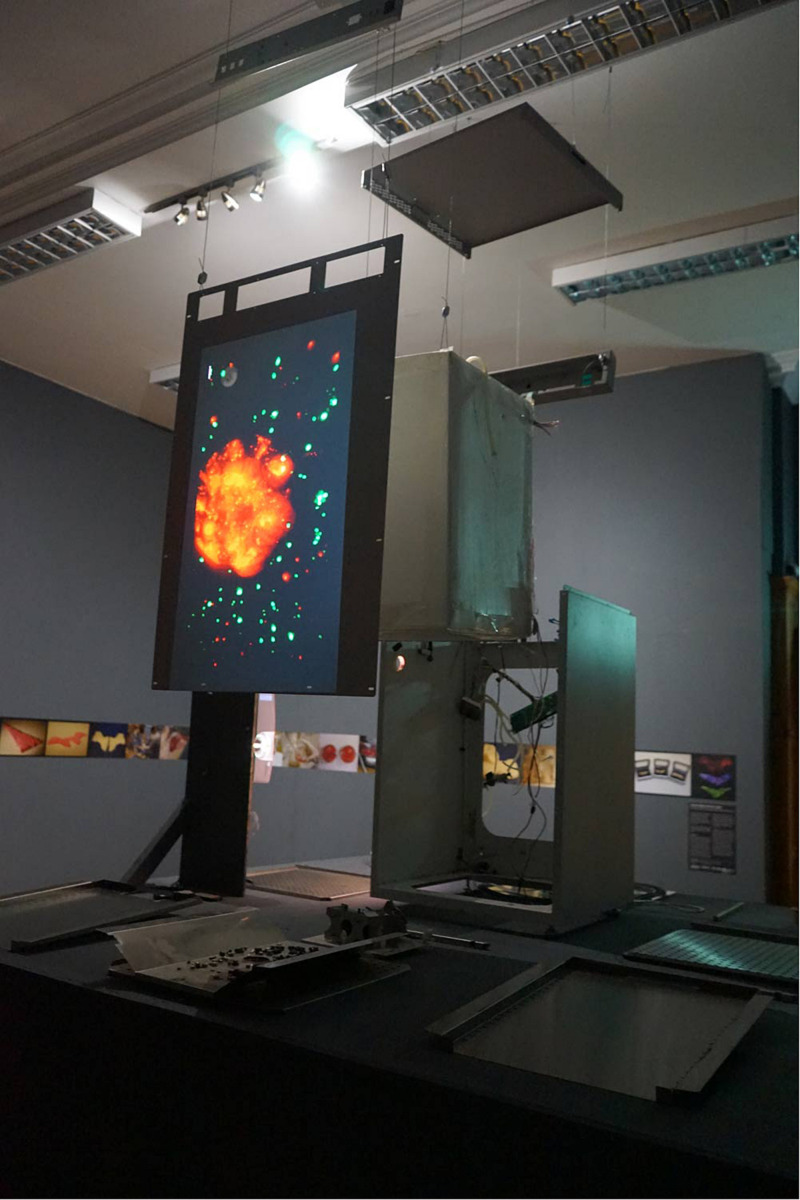
Work-in-progress installation of “Crossing Kingdoms” at Summerhall, Edinburgh.

“Crossing Kingdoms” facilitated such recontextualizations by collaborative wet-lab work between artists and synthetic biologists. The bio-artists and synthetic biologists worked together both to develop fusion protocols and to identify strategies for imaging and exhibiting that work. An artwork-in-progress was exhibited as part of the 2018 Edinburgh Science Festival, involving videos and still images from microscopy work projected onto a deconstructed incubator (Figure 2) (The Tissue Culture and Art Project, 2018). Scientific posters were also presented at two international synthetic biology conferences (Cachat et al., 2018; Smith et al., 2019) and scientists, social scientists, and artists together comprised a panel at an art conference (Bates et al., 2018).

### Approach to Cell Fusion

We employed a synthetic biology approach to engineer fusion between mammalian and yeast cells, rather than chemical methods often used to produce hybridomas (e.g., polyethylene glycol). We used a mammalian cell line, reported previously, expressing a fusogen on their surface, endowing them with fusogenic properties (Cachat et al., 2014). Human embryonic kidney cells (T-Rex 293) were engineered with p14FAST, a reptilian reoviral protein that induces cell–cell fusion by creating pores at contact sites between apposed cell membranes (Clancy et al., 2010). When grown in adherent, confluent cultures in the presence of a transcriptional inducer (tetracycline), these cells fuse with contacting cells to create large multinucleated cells. We used this cell line (clone THFU-10, also expressing the fluorescent reporter mCherry) to drive fusion between mammalian and yeast cells (*Saccharomyces cerevisiae*, also expressing GFP). However, *S. cerevisiae* possesses a thick polysaccharide cell wall that prevents the plasma membranes of both species from coming into contact with one another. To allow fusion, we prepared spheroplasts from yeast as described by Brown et al. (2017) and co-cultured them with THFU-10 cells under tetracycline induction for 3–10 h, as described by Cachat et al. (2018). Various microscopy techniques were used to image inter-species fusion events: fluorescence and confocal microscopy, as well as coherent anti-Stokes Raman (CARS) microscopy.

### Approach to Responsible Research and Innovation

Methods for “doing RRI” abound (e.g., Fisher and Schuurbiers, 2013; How to design a RRI-oriented project proposal - RRI Tools, 0000; Stilgoe et al., 2013). These are often protocols designed to ensure that reflection happens, by someone, at some point in scientific work. In practice, these protocols often define RRI so narrowly that little space is made for unscripted responses. In contrast, in this project, the social scientists experimented with the boundaries of RRI to question whether such a narrow definition of RRI is in itself responsible. We enacted a broader vision of RRI by cultivating an experimental space to enable alternative thinking—thinking that accounts for different sets of values or imagined futures that may otherwise be taken for granted, that may be routinely be limited by institutional norms but are fostered by literally or figuratively working in a different space. This project afforded several such spaces: the lab, which became a different space when artists and biologists worked together; an art conference, which became a different space through the presence of biologists; exhibition spaces, where audiences are encouraged to think differently about biology; and what we might call the epistemic space of the project, wherein researchers have been prompted to think differently as part of an atypical, experimental collaboration (Calvert and Schyfter, 2016). Consequently, both the collaborators and those around us have been challenged to rethink expectations about research process and outputs.

The social scientists’ role in this experiment is to observe where and when stereotyped ways of doing are emerging and to invite new ways to disrupt them, to facilitate conversation, and to apply social science analytics to project processes in the broader social contexts for biology. For example, the project raises questions about who is given social license to operate risky technologies and how we live with the “dragons” we create, with public exhibitions beginning conversations that feed back into ongoing research. Importantly, the social scientists’ own stereotyped thinking has been disrupted by the artists and biologists. We have all, in a sense, “done RRI” with and for each other.

## Has the Project Succeeded in Producing Cross-Kingdom Fusions?

“Crossing Kingdoms” has cultivated ample cross-kingdom contact, but cross-kingdom fusion is less certain. We were unable to distinguish biological fusion events from close contact events with the microscopy techniques we used. Work to confirm fusion is ongoing. Yet, identifying what constitutes fusion is a fundamental problem. Is it enough for the cell membranes of two cells from different kingdoms to fuse? Do their nuclei or genetic material need to interact? What if the larger cell engulfs the smaller cell, which remains visibly intact in the larger cell’s cytoplasm? Does the resulting fused entity need to remain alive? For how long? By what measure of aliveness? (Emerson et al., 2017) Does it need to consume energy? To reproduce? Not to self-destruct? Fusion events have been documented by demonstrating DNA uptake by mammalian cells, but yeast in these scenarios is solely a delivery device and ceases to be of interest after it has delivered its payload—not a cell–cell fusion accounting for the fate of both cells. A surprising amount of fuzziness exists around what exactly “fusion” is.

Similar fuzziness exists around disciplinary fusions. Is this project interdisciplinary (working across disciplines), transdisciplinary (transcending disciplinary boundaries), or merely multidisciplinary (involving several disciplines)? We think that this is the wrong question because it focuses on disciplines rather than on people. At present, the project’s impacts largely derive from enabling unexpected cross-disciplinary meetings. This artist-led project has been taken by both scientists and social scientists to synthetic biology conferences in poster form (Cachat et al., 2018; Smith et al., 2019). Two synthetic biologists based in the United Kingdom came to an art conference in Australia (Bates et al., 2018). Artists, synthetic biologists, and social scientists are all co-authors of this paper. We have jointly been prompted to reflect on our roles, what we are doing, and how our work may reshape others’ expectations. A more diverse, reflective, and creative range of *conversations* have thus taken place in the spaces that we and project outputs have occupied.

Yet while we have successfully fused our diverse goals and perspectives into a single project, we each retain our disciplinary interests and identities within the project. Is this fusion? We have not followed one ideal of “ontological” interdisciplinarity, in which researchers are individually freed from ordinary disciplinary constraints in pursuit of a common problem. However, one additional reason why this collaboration “works” is that we are each already interdisciplinary in our individual work. The artists are accustomed to wet-lab work. The social scientists work in science and technology studies, a highly interdisciplinary field. The scientists work in a synthetic biology center where lab teams collaborate with diverse researchers including artists and designers. Our backgrounds are important because we all be flexible in defining how this project matters on our own terms, such that our disciplinary territories can overlap without merging completely. While certainly not the only way to configure art-science or other interdisciplinary collaborations, our approach meant that we had less initial work to do in establishing common ground. Like both RRI and fusion, interdisciplinarity ends up meaning many different things in practice (Frodeman, 2017).

## Has the Project Succeeded as RRI?

Responsible research and innovation is often implemented as a checklist for researchers to demonstrate reflection on potential implications of their research. In practice, “responsibility” often involves demonstrating potential public benefits, and a relatively narrow range of potential benefits become routinely cited. We think that it would be irresponsible to use RRI to reinforce narrow expectations about what scientific research should do. By beginning as an artistic exploration and not expressly as an RRI intervention, this project has avoided being dominated by ethics and safety questions that often exclude discussions about other social dimensions of science and technology. Ethics and safety are important conversations, but not the only important conversations, and if we always direct attention to ethics and safety those other conversations may never happen. At no point have our discussions revolved around the ethics of engineering mammalian cells. Instead, we have considered what it means for scientific research to be useful, in addition to our initial questions about novel living things constructed through biotechnology.

“Crossing Kingdoms” employs a laboratory technique that had not been useful for biological research objectives. Cells expressing the snake virus-derived surface protein merge cell membranes with other cells reliably and efficiently (Smith et al., 2019), but the lab had no interest in fusing cells in this way. This seemingly useless system became useful when the artists introduced their desire to create cross-kingdom fusions. The artists describe their work as deliberately “useless,” in that their goal is not to create a knowledge product or solution, but to create artworks designed to be provocative rather than practical. The technique has thus become useful all over again to ask: what is synthetic biology or biotechnology good for? Coming full circle, this particular artistic exploration has also renewed the scientists’ exploration of this technique’s possible scientific uses.

**FIGURE 3:**
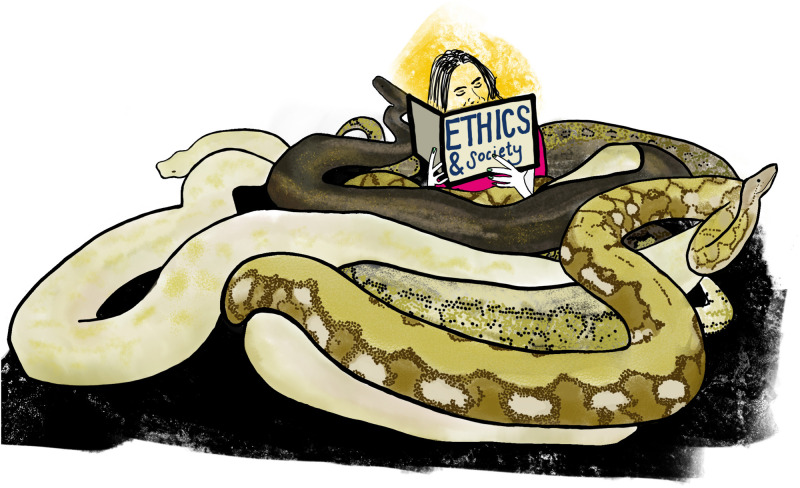


We argue that “Crossing Kingdoms” succeeds as an experiment in RRI in part precisely because it employs an inherently risky technology (Figure 3). When induced with tetracycline, THFU-10 fusion cells may potentially fuse with any cell membranes, including cells of living humans. Rigorous precautions must be taken when handling these cells. Using such a potentially dangerous system for art might seem irresponsible, even though similar risks are routinely taken in biomedical and other scientific research. The project complied with all health and safety guidelines that typically apply in such cases. No risks were taken beyond those usually involved in scientific research. This system is thus uniquely well suited for an experiment in RRI, as it raises the question: why should research for art be considered less worth entailing risks than research for other purposes?

It might be argued that “Crossing Kingdoms” is not a very provocative experiment because cell fusion (though not our novel, to the best of our knowledge, approach) is routinely performed. However, the artistic work is not intended to be *speculative*; they are not intended to depict something that viewers are invited to imagine being part of the future. Rather they are *reflective*, presenting something that recently happened in a real lab. RRI that addresses potential, often highly speculative futures of present science, avoids asking questions about real present events. The focus on current science is therefore a feature, not a bug, in our experimental design, allowing us to talk about social dimensions of science actually happening today.

## Concluding With More Questions

Cross-kingdom fusion raises ontological, ethical, and aesthetic questions. How do multi-kingdom cell fusions challenge current categories and understandings of life? Where do they belong within biological and cultural taxonomies? How does their existence impact environments and societies? Is scientific research conducted for art less valuable than scientific research conducted for other purposes? What can and should “responsibility” entail in practice? As is so often the case in research, our investigation has led to more questions than answers. However, we believe that generating questions is the point of research, not a mere by-product or indication of failure. When questions intersect with different value systems, as is inevitably the case in interdisciplinary research, questions are more valuable than answers because questions generate space for the articulation of more diverse voices than just our own.

## Data Availability Statement

The datasets generated for this study are available on request to the corresponding author.

## Author Contributions

ES, TB, EC, JC, OC, RS, and IZ designed the project. TB, EC, OC, LN, and IZ conducted lab work with assistance from SR, TB, and OC. IZ prepared art installations with assistance from EC, LN, and SR. ES wrote the manuscript with substantial contributions from TB, JC, RS, and IZ.

## Conflict of Interest

The authors declare that the research was conducted in the absence of any commercial or financial relationships that could be construed as a potential conflict of interest.
